# Detecting fecal microbiota transplantation–associated infection transmission using shotgun metagenomic sequencing and clonality analysis

**DOI:** 10.1017/ash.2023.345

**Published:** 2023-09-29

**Authors:** Emma Briars, Mohamad Sater, Nicole Billings, Ian Herriott, Emily MacLeod, Miriam Huntley

## Abstract

**Background:** Fecal microbiota transplantation (FMT) is a widely used modality for safe and effective treatment of recurrent *Clostridium difficile* infections, and FMT is being explored for the treatment of additional indications including gastrointestinal diseases and neurological disorders. Although microbiota-based therapies like FMT utilize rigorous donor screening procedures, these procedures are limited in resolution and scope, and there remains a risk of transmission of FMT-associated infectious agents from donor stool to a FMT recipient. Critically, these health concerns led the FDA to issue a 2019 safety alert for the transmission risks associated with FMT and to update its guidelines for screening and reporting. In a suspected transmission event, there is uncertainty around the source of infection; thus, methods are needed to rapidly determine whether a patient’s infection is linked to the donor stool product. **Methods:** Here, we developed a laboratory service sequencing and bioinformatics pipeline within our CLIA-certified laboratory for investigating suspected FMT infection transmission by measuring genomic relatedness. Our pipeline performs deep sequencing of a metagenomic sample, whole-genome sequencing (WGS) of an isolate derived from the implicated patient infection and determines the genomic relatedness between the 2 using a SNP-based analysis. The workflow was validated in silico with synthetic metagenomic samples spiked-in with WGS of clinically relevant isolate strains at varying abundance. **Results:** The sample and sequencing library preparation workflow was optimized across a panel of metagenomic and mock fecal microbiome samples demonstrating reproducible and reduced-bias sequencing of metagenomic samples. Our pipeline demonstrates high sensitivity and specificity for clonality calls when a spiked in isolate genome achieves 5× depth for >50% of the genome. We also demonstrated an interplay between abundance rate and sequencing depth for determining a clonality limit of detection. **Conclusions:** Taken together, our pipeline represents a new method that can support the clinical efforts of FMT and other microbiota-based therapies. **References:** US Food and Drug Administration. Important safety alert regarding use of fecal microbiota for transplantation and risk of serious adverse reactions due to transmission of multidrug-resistant organisms. Rockville, MD: Food and Drug Administration, 2019. DeFilipp Z, Bloom PP, Torres Soto M, et al. Drug-resistant *E. coli* bacteremia transmitted by fecal microbiota transplant. *N Engl J Med* 2019;381:2043–2050.

**Financial support:** This study was funded by Day Zero Diagnostics.

**Disclosures:** None

